# Superparamagnetic Nanoparticles Targeting Brain Cancer: Innovations in Carbohydrate-Based Coatings and Magnetic Field Guidance

**DOI:** 10.3390/cancers18030419

**Published:** 2026-01-28

**Authors:** Ahmed Mahdi Abed Alobaidi, Vadim V. Kumeiko

**Affiliations:** 1 Faculty of Biomedicine, Far Eastern Federal University, 690922 Vladivostok, Russia; ahmedalnask@gmail.com; 2 A.V. Zhirmunsky National Scientific Center of Marine Biology, Far Eastern Branch of Russian Academy of Sciences, 690041 Vladivostok, Russia

**Keywords:** superparamagnetic iron oxide nanoparticles (SPMNPs), brain cancer, blood-brain barrier (BBB), carbohydrate-based coatings, magnetic targeting, glioblastoma

## Abstract

The effective treatment of aggressive brain tumors, such as glioblastoma, is critically hindered by the blood-brain barrier (BBB) and the non-specific clearance of therapeutic agents by the immune system. Superparamagnetic iron oxide nanoparticles (SPMNPs) offer a powerful theranostic platform, combining magnetic resonance imaging (MRI)-based diagnostics with therapeutic delivery and hyperthermia. However, their clinical translation requires sophisticated strategies to ensure precise delivery to the tumor site. This review examines innovative functionalization strategies to enhance the targeting and efficacy of SPMNPs. Specifically, it addresses the various strategies for coating magnetic nanoparticles with carbohydrates, including both covalent and non-covalent methods, and the subsequent functionalization of these glycoconjugates to exploit the unique biological environment of brain tumors. The use of glycoconjugates on the nanoparticle surface is a key strategy, leveraging the altered glycosylation patterns and overexpression of specific lectins on glioma cell surfaces to achieve highly selective cellular targeting. The review details the synergistic effect achieved by combining these functionalized nanoparticles with external magnetic field guidance. This combination provides a dual-action mechanism: the magnetic field actively guides the nanoparticles across the BBB and concentrates them within the tumor mass, while the carbohydrate coating ensures specific cellular uptake, thereby significantly improving local therapeutic concentration and minimizing systemic toxicity. The scope of this review includes the development and evaluation of carbohydrate-coated SPMNPs, outlining their optimized physicochemical properties for both in vitro and in vivo imaging and treatment of cancerous brain tissues. This comprehensive evaluation represents a critical advancement in biomedicine, aiming to improve the prognosis for patients with brain cancer through more precise and effective therapeutic interventions.

## Simple Summary

Brain cancer is highly challenging to treat because the blood-brain barrier, a natural shield, prevents therapeutic agents from reaching the tumor. This review explores innovative strategies utilizing tiny magnetic particles to overcome this challenge. We summarize and discuss how coating these particles with specific carbohydrates, which are selectively attracted to cancer cells, enables highly targeted drug delivery. This review also examines how the use of an external magnetic fields can guide these particles directly to the tumor. By analyzing the current state of this technology, we highlight its potential to create more effective and less toxic therapies, offering a comprehensive look at a promising new tool for the medical community in the fight against brain cancer.

## 1. Introduction

It has been historically viewed that the problems with nanoparticles in the treatment of brain cancer were primarily related to modeling: the transition of nanoparticles through the main barriers in the tumor mass was obviated either due to their physical properties or due to passive diffusion and retention in the tumor parenchyma [1]. However, these views have been incomplete and should include one more aspect: how to prevent nanoparticles from active uptake by the phagocytic immune system and the subsequent clearance in the liver and the spleen [2].

Commercially available nanoparticles show maximum accumulation in a tumor one hour after parenteral administration. SPMNPs are taken up from the blood stream by macrophages due to their large surface area, in effect a magnetically enhanced “EPR”-like mechanism. However, several days after administration they start to accumulate in the liver and spleen, and according to in vitro studies on different cells tested, they also exhibit severe cytotoxicity [3,4,5].

Glioma comprises about 30% of all brain tumors, of which glioblastoma is the most aggressive form. The heterogeneous nature of glioma contributes to the ineffectiveness of current treatment modalities [6]. The standard multimodal treatment for glioblastoma consists of maximal safe surgical resection, radiotherapy, and concurrent and adjuvant temozolomide chemotherapy [7,8].

Novel therapeutic approaches such as nanoparticle-based treatment are gaining increasing attention because of their unique physicochemical properties. SPMNPs have emerged as a complementary approach for glioma therapy by acting as an MRI contrast agent, a drug carrier, and a hyperthermia heat source [9,10]. However, the intracellular uptake and extravasation properties of the nanoparticles are hampered by non-specific protein coronas and the BBB [11].

Nanoparticle functionalization with a targeting carbohydrate-based ligand together with magnetic field guidance has recently been reported as an effective strategy capable of overcoming these limitations [12].

The use of nanoparticles in glioma treatment is being recognized as a novel strategy because of their potential as a contrast agent in MRI and as a system for site-specific delivery [13]. The application of ultra-small nanoparticles as a contrast agent for gliomas and for hyperthermia is also being considered. Recent reviews outline the most exciting applications of iron oxide nanoparticles focusing on magnetically targeted nanoparticles for both the hyperthermic heating of tumors and detection in MRI [14,15].

In fact, owing to the complexity of the central nervous system and other factors, the relative dissemination of these tumors is described in contrast to metastasis [16,17,18]. However, malignant transformation in other organ systems within the central nervous system can indeed lead to infectious behavior and occasionally hematogenous dissemination [19]. Chemotherapy that must cross the BBB is less effective in pure volume reduction than in other non-local CNS chemotherapy roles, including the temporary prevention of cardiac seizures and treatment of some infectious diseases at an experimental level [1,20].

The search for methods that allow the selective concentration of molecular species needed for diagnosis or therapy, or the penetration of these zones in a magnetic field gradient, has generated interest in the use of nanoparticles. When a nanoparticle or a therapeutic formulation contained within one is introduced into the bloodstream, its movement through the microvasculature is affected by interactions with the walls of red blood cells in the larger and faster capillaries. In these areas, SPMNPs can enhance deformation even in the presence of a weak magnetic field [21,22].

SPMNPs have recently emerged as a very interesting platform to develop new therapies for brain cancer. The main goal of our approach is to use magnetically responsive nanoparticles not only to effectively deliver a chemotherapy drug to cancerous areas but also to minimize potential side effects in normal tissues by increasing drug bioavailability and reducing deleterious systemic delivery [23]. Following efficient drug administration, it is essential that the nanoparticles are either eliminated or dissolved at the nanoscale. This step is crucial to prevent any potential disruption to the re-epithelialization process and to facilitate the formation of functional glial scars.

Currently, novel amorphous glycomaterials for SPMNPs are used in brain cancer research, including their ex vivo MRI detection and accumulation-enhancing magnetic field treatment [24].

Brain cancer claims about 150,000 lives annually in Europe, which is a major public health issue. So far there are a number of new methods that are created to enhance the penetration of chemotherapeutic drugs into the brain and the results are positive but overall results are not satisfactory. Micro-nanotechnology shows promise of innovating more and making use of the enormous potential of pharmaceutical therapy that can be applied to brain tumor [25]. SPMNPs are amongst the most interesting advances, showing considerable applicability in imaging procedures as well as therapeutics. After these functionalized SPMNP have been internalized by the target cells, they can be precisely directed to their ultimate destination by applying an external magnetic field, a process called magnetic targeting [26]. This targeting application is particularly important in application with brain tumors where precision is of the essence. In the recent past, it has been found that some polysaccharides that are produced by the different plants have a potential of preventing the proliferation of human brain cancer cells [27].

This review is focused on the development of iron oxide nanoparticles coated with carbohydrate-based biocompatible materials for brain cancer theranostics. Three innovative topics are addressed, including novel coatings, strategies for their targeting to brain tumors, and direct administration of nanoparticles into a mouse brain under the management of an external magnetic field.

## 2. SPMNPs: Properties and Applications

**Figure 1:**
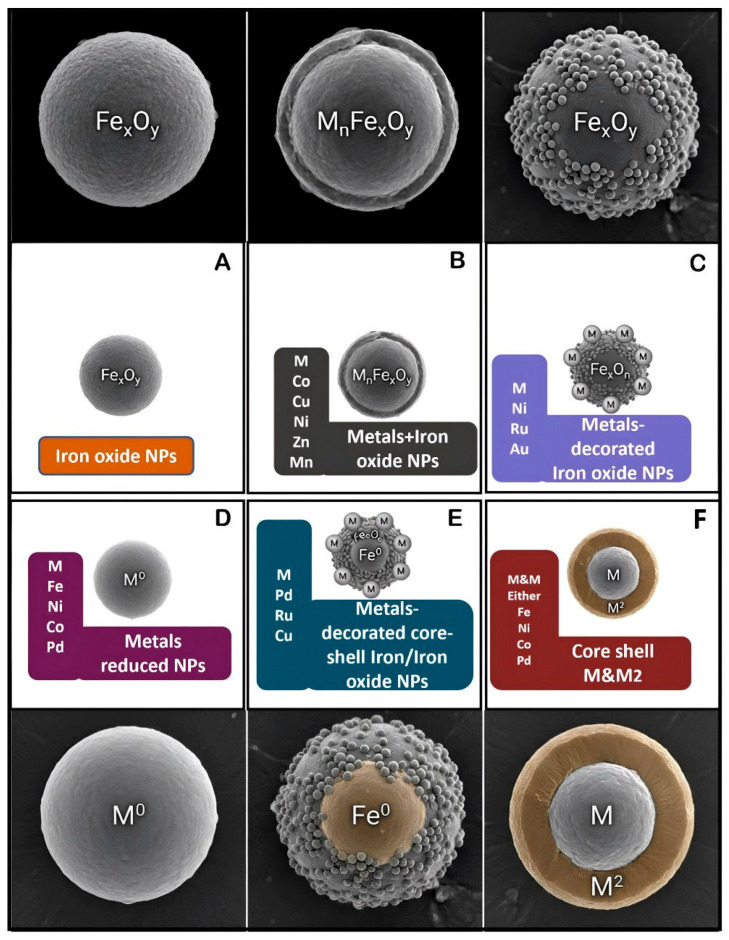
General mechanism of iron doped catalyst. (**A**) Iron Oxide NPs: This shows a spherical iron oxide nanoparticle (Fe_X_O_y_), which serves as a base material; (**B**) Metals + Iron Oxide NPs: This depicts a core-shell structure where a layer of metals (M_n_) is incorporated with iron oxide, forming Mn_X_Fe_X_O_y_. This process involves adding metals like Co, Cu, Ni, Zn, or Mn; (**C**) Metals-Decorated Iron Oxide NPs: This illustrates iron oxide nanoparticles (Fe_X_O_y_) whose surfaces are decorated with smaller metallic nanoparticles (M). The metals used for decoration can include Ni, Ru, or Au; (**D**) Metals Reduced NPs: This shows a solid metallic nanoparticle (M^0^), which is formed by reducing metals such as Fe, Ni, Co, or Pd; (**E**) Metals-Decorated Core-Shell Iron/Iron Oxide NPs: This shows a core-shell structure with an iron core (Fe^0^) and an iron oxide shell. The surface is further decorated with various metal nanoparticles (M), such as Pd, Ru, or Cu; (**F**) Core-Shell M&M2: This illustrates a dual-layer core-shell nanoparticle, with an inner core (M) and an outer shell (M^2^). The metals (M and M^2^) can be composed of materials like Fe, Ni, Co, or Pd.

SPMNPs possess unique physical properties that make them highly valuable for various biomedical applications. Specifically, their strong response to external magnetic fields allows their use as contrast agents in MRI and as vehicles for targeted drug delivery in magnetic field-guided cancer therapies [28,29]. The composition of these nanoparticles typically includes magnetic materials, such as iron, cobalt, nickel, or their respective oxides. To enhance their magnetic properties for applications such as MRI, these core materials can be further decorated with other metals, including nickel (Ni), cobalt (Co), manganese (Mn), palladium (Pd), and copper (Cu) (Figure 1) [30]. Iron and iron oxide-based nanoparticles are particularly effective for MRI because of their large magnetization under moderate magnetic fields, Repeat administration of SPMNPs contrast agents is currently considered to be safe for patients who require successive MRI investigations [31]. Nanoparticles smaller than 15 nm exhibit superparamagnetism [32], where magnetic dipoles align under an external field [33]. This collective magnetization disappears when the field is removed, leaving no residual stress or remanence [34,35].

### 2.1. Definition and Characteristics of SPMNPs

SPMNPs are one of the most promising and developed nanomaterials and currently the target of robust research and development as the therapeutic agent against different diseases in the context of nanotechnology and biomedicine. Superparamagnetism is a phenomenon that is a result of a combination of both small crystal/grain size and the existence of a magnetic ordering temperature [36].

If the size of a magnetic particle becomes below a critical limit, then its magnetic response has zero magnetic remanence, and the magnetic ordering temperature is less than absolute temperature. This produces single-domain behavior, the main characteristic of the superparamagnetic state [32,37].

Such particles exhibit zero coercivity, no hysteresis loop in the magnetization curve, and finite magnetic susceptibility at temperatures greater than absolute zero [38]. Thus, SPMNPs are not magnetized in the absence of an external magnetic field, which significantly enhances their utility as an optimal vehicle to transport therapeutics at both the cellular and tissular levels. This characteristic is particularly advantageous, as it ensures that there are no aggregation effects occurring, allowing for a smoother and more efficient delivery of therapeutic agents [39].

These particles can be functionalized with therapeutic molecules, and they will accumulate in neoplastic cells or tissues by applying external magnetic fields [40]. Furthermore, the particles can also reach the inner part of the tissue by applying an alternate high-intensity magnetic field, and their superficial modification can be controlled by modifying the duration and intensity of exposure to the same high-intensity magnetic field [41]. The aim of this review is to focus attention on the SPMNPs in a very specific biological application, i.e., bringing functionalities able to interact with neoplastic cells, such as carbohydrates, to bring the SPMNPs into the cancer cells at the same time as they are guided into the cells by exposure to high magnetic gradient events without inducing cytotoxic effects, unlike other proposed coatings [42].

### 2.2. Synthesis Methods for SPMNPs

**Figure 2:**
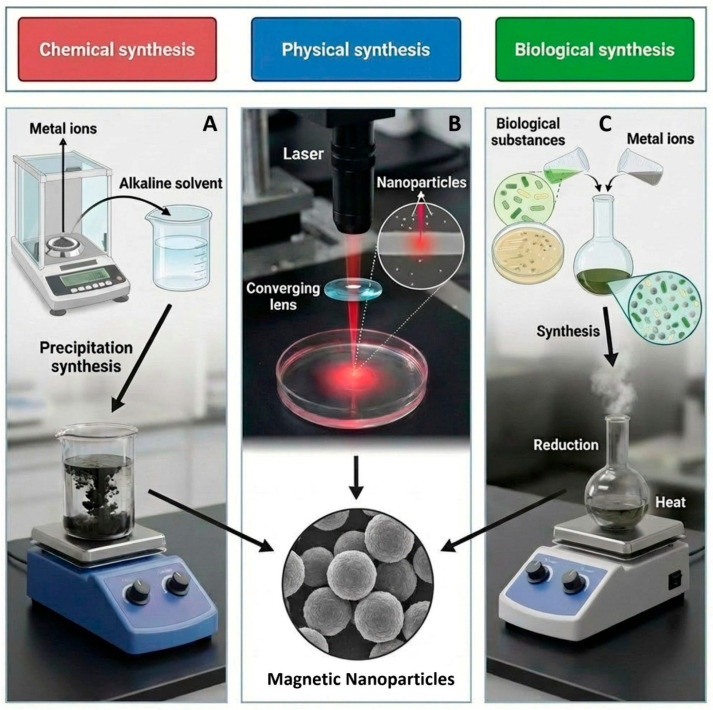
Synthesis methods of super magnetic nanoparticles. (**A**) Chemical Synthesis: Involves combining metal ions and an alkaline solvent, followed by precipitation synthesis to yield magnetic nanoparticles; (**B**) Physical Synthesis: Utilizes a laser focused by a converging lens to ablate a target material, resulting in the formation of nanoparticles; (**C**) Biological Synthesis: Combines biological substances and metal ions, followed by a synthesis and reduction process, often involving heat, to produce magnetic nanoparticles.

The synthesis of SPMNPs can be classified as a chemical or physical reaction between the precursor, whereas the core and shell of nanoparticles are formed with the help of stabilizing agents, forming a final composite [43,44]. Physical methods have been increasingly gaining traction in recent years due to the effective production of smaller-sized nanoparticles that exhibit a controlled shape [45]. The advancements in these techniques allow for precise manipulation of the nanoparticle’s characteristics (Figure 2) [43].

Other notable methods for synthesizing small particle metal nanoparticles (SPMNPs), particularly where the function of high-energy reduction of the metal salt plays a significant role in the core-shell structure of the resulting particles, may encompass a diverse range of approaches [46,47]. These approaches can include the careful mixing of various reagents, the innovative technique of microwave synthesis, the utilization of Co self-combustion processes, the versatile sol-gel method, and several others that continue to emerge in the field. Each approach contributes uniquely to the development of nanoparticles with desirable properties for various applications, underscoring the importance of ongoing research in this dynamic area [48]. Nevertheless, the use of an appropriate capping agent is necessary to prevent the agglomeration of the final nanoparticles, which can cause severe problems with particles’ applications in both commercial and research scenarios [49].

The control of the amount of coating agent on the particles surface is also another important issue in the synthesis of magnetite [50]. The necessary amount of material in form of synthetic carbohydrates is a critical parameter to avoid aggregation of the NPs and the dispersion and stability of the particles in water. In contrast, in case of Fe_3_O_4_/Fe_2_O_3_, the surface magnetism and susceptibility also depend on the capping agent, and higher properties in imaging methods are needed [24,51]. Moreover, biological compatibility, which should be enhanced by raising the amount of saturation magnetization in a bid to produce a more effective separation to prevent aggregation of the particles or any other problem that may facilitate the loss of the magnetism by the particles when transferred to the biological medium are of paramount importance [52,53]. Lastly, an easy, quick, and cost-effective synthesis process is a fundamental consideration in the manufacture of magnetite particularly in the full-scale industrial synthesis [54].

### 2.3. Functionalization Strategies for Targeting Glioma Cells Using Carbohydrate-Based Coatings

As presented earlier, glycoconjugates hold the most promise in providing efficient multicellular targeting to SPMNPs, and we are witnessing intriguing bio functionalization strategies that are leading to this goal [55].

Here, we review briefly the critical findings in this area related to exploiting the carbohydrate-specific interactions that the nanoparticles will display on their surfaces, prior to reviewing current descriptions of the artificial tumor microenvironment, quintessential to further development of nanoparticles as efficient targeting agents for glioblastoma cells [56]. On surface of targeted cells, lectins have unique and important binding roles among the class of other proteins involved in cell-cell and cell-matrix adhesion and signaling [57,58].

It has been established that bio conjugation of targeted particles with currently available lectins will (a) minimize the binding to unwanted target cells, which would be the healthy brain tissue cells that lie in very close proximity to the glioma brain cancer cells; and (b) specifically target glioma brain cancer cells that express a unique glycosylation pattern on their cell surfaces with overexpression of the glycan ligands of the bio conjugated lectin(s). Although this is a major advantage for lectin-based targeting strategies, researchers so far have undertaken a very limited analysis of glioma carbohydrate specificity as revealed by cell-based glycomic analyses [59,60].

The other important question is the dependence of the targeting strategies on the presence of the cancer-specific glycans on the structure of the glycoconjugates, as the majority of the methods developed to date in the synthesis of carbohydrate-functionalized SPMNPs have not determined whether these ligands will be available to facilitate the flow of the micellar or vesicular delivery and colonization of the complex physiological environments [61]. This important structure information should consider a number of factors. As an example, it is necessary to refer to the requirements to graft the ligands to the surface of SPMNPs in a meticulous manner [62]. Moreover, it is necessary to consider whether the glycan-functionalized nanoparticles so produced can be effective in replicating the glycocalyx that naturally occur on living cells. This ability is essential to enable them to bind effectively to the targeting receptors with the spatial orientations as well as geometrical configurations needed to facilitate effective interactions.

The knowledge of these complex interactions is important in creating the best nanomaterials in therapy. It is also of major importance to provide a comprehensive characterization of the glycan expression on cancer cell-surface hydrophobic interactions among the glycan ligands and the lectin molecule present in the microenvironment of the targeted cells because accessibility of glycan by the complex and heterogeneous microstructure of the healthy tissues is possible due to the heterogeneous mixtures of the proteins, cells, and the extracellular matrix [63].

The use of glycan-functionalized nanoparticles can lead to complex and unintended consequences that affect targeting specificity. These outcomes include the potential modification of cellular bio-adhesive properties and interference with therapeutic agents designed to act on glycan levels. Such interactions can alter targeting efficacy and impact the progression of the cancer.

Consequently, it is imperative to expand research efforts aimed at elucidating these distinct interactions. A deeper understanding is necessary to account for the constantly evolving nature of cancer biology and to achieve more precise control over the nanoparticle-cell targeting process through the development of advanced materials and novel guidance strategies [64].

The pectin-coated SPMNPs are an emerging and potentially highly promising technology that can lead to better targeting of brain cancer cells, especially glioma cells [65].

This novel approach takes advantage of the specialized and distinct interactions, which exist between individual glycan structures that exist on pectin and the glycan-binding proteins, which appear on the surface of glioma cells. The essential interactions are crucial because they help in delivering different therapies efficiently and targeting them to the center of the tumor site, and thus have the potential to enhance treatment results of patients who are already facing complexities of dealing with brain cancer. This approach, which targets the improvement of the accuracy of drug delivery systems, does not only promise to expand the therapeutic effectiveness, but also seeks to reduce the side effects, which is a notable step in the treatment of gliomas and other malignant brain tumors [66].

Magnetic nanoparticles with polymer coating have become a topic of great interest to researchers in the fields of Nano-biomedicine and basic biomaterials science. By having imaging properties with external stimuli responsiveness when covered or encased in polymers, these theranostic magnetic nanoparticles are efficient platforms not only in the delivery of various drugs and therapeutic genes, but also in the imaging of their presence. Although the production of polymer-coated magnetic nanoparticles has been largely successful in the last decade, their use has largely been limited to imaging. This paper dwells upon the synthesis and use of polysaccharide-coated magnetic nanoparticles, and the authors are interested in the application of these materials in sophisticated imaging approaches and gene delivery systems [67].

Various studies employing different polysaccharide modifications and payload have been presented, as illustrated in (Table 1), to establish the different uses of polysaccharide materials in drug delivery and therapy. The application of alginate, for instance, has been explored for myoblast cell line studies in vitro, employing APA-modified alginate [68]. The application of chitosan modified with CTX and PEG could, according to studies conducted in mice, provide drug delivery for brain tumors [69]. The application for in vitro hematopoietic and neural progenitor cell targeting has been demonstrated by dextran conjugated with FITC-derivatized Tat peptide [70]. The application of hyaluronic acid and dopamine was demonstrated in cell line studies involving HCT116 and NIH3T3 with implications for medication efficacy [71]. The use of heparin in anti-tumor therapy was demonstrated in SCC-7 tumor-bearing mice employing gold-deposited glycol chitosan [72]. The application of mannan, specifically carboxylic-modified mannan, was demonstrated for subcutaneous injection delivery in rat studies [73]. Curcumin is wrapped inside a pectin capsule, which possesses anti-cancer activity against various in vitro, as well as in vivo, cancer models for its anti-cancer properties [74]. L929 cells, as well as KB cells, have been employed for the studies of hyperthermia to evaluate the hyperthermic property of Pullulan [75]. Finally, starch biologically modified with PEG was investigated for the delivery application of anti-tumor drugs for 9L-glioma brain tumors in the male Fischer 344 rat model [76]. This comprehensive review emphasizes the versatility of polysaccharides, which have been employed in targeted delivery designs using various model systems, including in vitro cell cultures through to in vivo models.

### 2.4. Current Applications in Cancer Therapy and Diagnostics

A critical interpretation from this review is that SPMNPs—when conjugated with targeting ligands, specialized coatings, or functionalized with therapeutic agents—represent the most prevalent strategy for detecting, diagnosing, and managing cancer [9]. To enhance the prospects of successful clinical translation, each specific challenge in treatment must be addressed to enable more precise and discriminative therapies [77].

Over recent decades, cancer therapy has evolved through numerous experimental approaches aimed at inducing apoptosis. These include early surgical resection, conventional chemotherapy (often toxic and non-specific), radiation therapy, and more advanced modalities such as cytokine therapy, cancer vaccines, antibody-based targeting, gene therapy, antibody-drug conjugates, and oncolytic viral therapy [78]. While significant progress has been made in these areas, the goal for oncologists and pharmacologists remains to develop more effective, benign, and innovative therapies that ensure safe, cell-specific targeting for severe diseases [79].

Within the specific context of targeting angiogenic abnormalities, experimental magnetic targeting has validated the hypothesis that tumor-associated blood vessels can be exploited for site-specific drug delivery. This approach has demonstrated the effective migration of nanoparticles and shows promise for achieving more sustained therapeutic effects in cancer treatment [80,81]. The biological accuracy of these experiments underscores the credibility of applying this methodology *in vivo* and highlights the potential of magnetic nanoparticles to specifically target and induce tumor cell death [82].

Furthermore, the dual application of SPMNPs for concurrent imaging/diagnosis of the tumor microenvironment and for monitoring therapeutic progress in clinical settings confirms their significant relevance. This utility is demonstrated across a wide range of precise and necessary experiments [9].

## 3. Targeting Mechanisms for Glioma Cells

A carrier molecule targeting a receptor on the glioma cell surface initiates the most conventional active targeting approach: receptor-mediated transcytosis mechanism [83]. To accomplish this process, a brain-blood barrier-crossing molecule must be selectively expressed in the glioma endothelium and the glioma cells [84]. In the development of a suitable BBB-crossing carrier, a monoclonal antibody is capable of vital site-specific recognition and the provocation of focused delivery [85].

Carriers for entry to the endothelial and subsequent glioma transcytosis include the transferrin receptor, low-density lipoprotein receptor, glucose transporter, and folate transporter [86]. However, these receptors can also be expressed non-specifically in other cell types, including red blood cells, further increasing the perfusion risk. The lower capillary tolerance can aggravate side effects related to these carriers [87].

Glioma cells can also possess a functional mechanism for drug rejection, yet the rejection of the drug is a prerequisite for effective glioma therapy [88]. The glioma BBB is dynamic due to the existence of a high fraction of fine blood vasculature. When the tiny vasculature is blocked, the invasion of glioma is impossible. On the other hand, a smart carrier molecule makes it easier to reach glioma cells through certain receptor-mediated signal pathways [89].

Since the internalization capacity of the carrier-specific ligand-glioma cell regulator binding is still being optimized and polished, the transmission process could cause a quick internalization as a result of some cycle studies and experiments. It is due to this phenomenon that the mechanisms that are involved in these processes [90] are important to understand. Also, dispersed multijunctional carriers play a major role in targeting brain cancer due to the use of an advanced multi-targeting strategy, which aims at engaging multiple types of CD44 receptors [91]. The binding potential can result in a wider and more versatile targeting spectrum by allowing different attractive moieties to be combined together as well as seeking to reduce any interactions that may be occurring with other non-target cell types. This not only enhances specificity, but also increases the effectiveness of treatment modes in general in the case of brain cancer [92].

### 3.1. Overview of Glioma Cell Biology and Microenvironment

Gliomas are the most common primary brain tumors in adults, showing the highest degree of malignancy and invasiveness [93]. Tumor heterogeneity, tumor microenvironment, and distant invasion observed as spreading throughout the brain are distinctive features of glioma [94]. Intrinsic and extrinsic properties and molecular changes in glioma-associated brain cells, including macroglia, neurons, endothelial cells, pericytes, brain-resident microglia, and circulating monocytes, affect tumor resistance, angiogenesis, infiltration, and invasion that support glioma progression [95].

Consequently, cells near glioma are not simply innocent bystanders but play active roles in tumor biology [96]. Although many scientific findings revealed that tumor cell biology or microenvironment supports insights into tumor development and progression, a completely satisfactory therapeutic modality has not yet been achieved [1,97]. With advances in cancer research, a myriad of new, attractive therapeutic targets has been discovered that researchers hope to exploit to battle cancer [98].

Glioblastoma (GBM) is the most aggressive and deadly type of primary brain cancer classified as a grade IV glioma, presenting rapid and deadly proliferation [99].

Approximately 21,000 Americans are diagnosed with glioma and over 13,000 of them with GBM each year, but patient survival rates are tragically low even with treatment combining neurosurgery, radiation therapy, and chemotherapy [100,101]. Consequently, targeted treatments for glioma are actively being explored and developed in cancer research. To develop the perfect, patient-customized drug, basic and clinical researchers are collaborating in a multidisciplinary approach, which strengthens the bridge between bench and bedside [102].

### 3.2. Mechanisms of Targeted Delivery (Passive vs. Active Targeting)

The delivery of SPMNPs to the tumor site can be achieved by employing their unique properties for imaging or for targeted hyperthermia. Nanoparticles must navigate through different barriers, including endothelial layers and phagocytic cells or the penetration through extravascular matrices [103,104].

The active targeting of tumor cells requires the strong manipulation of tumor form or cell membrane structure, and the enforced permeability for the nanoparticles is needed [105]. However, it is possible to select a specific type of nanoparticle recognizing the molecular signatures of tumor cells [106].

Hydrophilic polyethylene glycol and carbohydrate coatings fundamentally change the properties of magnetite nanoparticles by reducing non-specific uptake and improving biocompatibility [107]. These materials can be used for tumor targeting under the action of external magnetic fields, causing spatial localization, aggregation, or enhanced cell permeability.

In general, magnetic field guidance helps to employ low-concentration nanoparticles with maximum tumor specificity [108]. The exposure of targeted nanoparticles to an external rotating magnetic field provides uniform spatial heating of tumor tissue without causing temperature elevations in normal mouse organs [109].

The application of iron oxide nanoparticles coated with sialic acid or fucoidan for therapy or MRI of brain cancer does not disturb human red blood cells, and the heparinized parenteral administration does not alter prothrombin and activated partial thromboplastin time in mice [110]. The use of glycan-coated nanoparticles in combination with magnetic field guidance is new, and their interactions with blood cells are completely unstudied [111].

### 3.3. Role of Ligands, Including Antibodies, in Enhancing Specificity for Glioma Targeting

Coupling of SPNs with different non-toxic coatings, bearing single or combined targeting ligands, can provide:

A very high accumulation of SPNs in orthotopic models of GBM via transport across the BBB due to the enhanced permeability and retention effect and surface charge-independent interactions with endothelial and malignant cells [112].

Significant internalization by glioma cells in vivo via propitious epidermal growth factor receptor-mediated mechanisms and high surface avidity [113].

Efficient site-specific retention in the tumor microenvironment due to unique anti-inflammatory and cytokine expression-modulating properties of coatings and antibodies [114].

Now, especially beneficial effects of surface glycans, even when SPN coatings are already established, should be emphasized. Glycans should contribute to SPN functionalization as imaging agents and drug coatings in addition to lipids, not only for increasing SPN affinity towards glioma [115]. This can be achieved through the use of glycans providing unique GSC-glycomic patterns specific for GBM, but absent in normal brain cells and blood vessels [116]. Glycans and glycoconjugates can be designed for increasing in vivo SPN performance; the overall contrast-enhanced image brightness, and the accuracy of drug release at the targeted site, as the highest therapeutic indices with the least adverse effects can be reached [117].

Ideally, for carbohydrate-based SPN finishing, the crucial need is given for transforming features after newly recruited SPN coatings (binding with pathologically altered membrane proteins to improve the affinity of penetration and durability during prolonged contact with the GBM microenvironment) into gradual, response-to-stimuli changes conducting drug release, tolerating off-site protected circulation of SPNs [118].

## 4. Carbohydrate-Based Coatings for Nanoparticles

By incorporating magnetic nanoparticles into MRI contrast reagents and enhancing their accumulation in pathophysiological regions, magnetic field-guided molecule delivery can increase MRI sensitivity while decreasing contrast agent dosages [40]. Positively charged SPMNPs targeting negatively charged cell surfaces’ sialic acids display high specificity for targeted cellular accumulation [119]. Specifically, a combined monosaccharide with multiple carboxylic groups can serve as a “dual target” to first form a stable monolayer, followed by covalent linking to further keep attached to a magnetic nanoparticle covering as the particle enters a cell surface’s contact position [120].

The SPMNPs selectively targeted MCF-7 cancer cells that repurposed those carbohydrate-coated nanoparticles [121,122]. A glucose-coating carboxylic acid-surface modified monolayer was then anchored to the nanoparticle surface by carboxylic acid-based covalent esterification or amide binding to form carbohydrate-coated nanoparticles [123]. Isomers were recognized depending on whether the modified dextran presented carboxylic acids on a terminal glucosyl residue or a second glucosyl residue within the saccharide chain [124]. Overall, they could indeed drive nanoparticles to focus right in proximity to the magnetic-assigned target cell surfaces [125].

(Table 2) aptly shows the variability present in polysaccharides and how they differ in origin as well as the physicochemical characteristics responsible for their function in nature. Indeed, these biopolymers are widely present in nature—vegetable sources like Alginate and Starch, Algae like Alginate; Animal sources like Chitosan and Heparin; and microbial origin like Dextran and Pullulan. A crucial feature of these polymers is their electrical charge, which significantly impacts their molecular interactions. For example, anionic polysaccharides like hyaluronic acid and pectin are negatively charged due to their carboxyl and sulfated hydroxyl groups. In contrast, chitosan is positively charged because of its protonated amine groups, while others like mannan and starch are neutral, being composed mainly of uncharged hydroxyl groups.

### 4.1. Introduction to Carbohydrates as Biocompatible Polymers and Their Properties

Research into the design and synthesis of biocompatible polymers represents one of the most active areas in polymer chemistry [126]. These tailored polymers exhibit exceptional properties, making them highly functional as biomaterials in biotechnology and medicine [127].

Among the various biocompatible polymers developed, hydrophilic carbohydrates have attracted significant attention due to their intrinsic multifunctional nature. Most efforts have focused on the polymeric architectures of naturally existing polysaccharides or nature-inspired synthetic carbohydrates [128].

The excellent physicochemical properties, functionalities, and biocompatibility of carbohydrate polymers establish them as unique and promising materials for a wide range of biomedical applications. Composites based on hydrophilic and biodegradable carbohydrate polymers have been developed as effective drug delivery systems capable of encapsulating high payloads of hydrophilic drugs [51]. By adjusting the chemical and physical properties of these polymers, a controlled release profile can be achieved through careful design of the polymers and their formulation methods as conjugates or complexes [129].

Well-defined polymeric amphiphiles have been specifically designed to formulate hydrophobic anticancer drugs. By modifying these polymers with a precise number of hydrophobic groups, the resulting amphiphilic carbohydrate polymers can self-assemble into stable micellar nanostructures in aqueous environments. These self-assembled nanostructures offer several significant advantages as drug delivery systems, including enhanced drug solubility, prolonged circulation half-lives, improved stability, and the potential to create stimuli-responsive nanocarriers for triggered drug release [130,131].

Recognized as inherently biocompatible polymers, carbohydrates possess distinctive properties that make them excellent candidates for medical applications, particularly in targeted treatments for conditions such as brain cancer [132]. For instance, coating SPMNPs with pectin—a type of carbohydrate—enhances their biocompatibility and stability, facilitating their use in therapeutic interventions. Pectin’s natural affinity for biological interactions may further improve the efficacy of these nanoparticles in delivering targeted treatments [133].

### 4.2. Description of Methods for Coating SPMNPs with Carbohydrate Materials

The coating of SPMNPs with carbohydrate materials is a critical step that significantly enhances their biocompatibility, colloidal stability, and functional efficacy for diverse biomedical applications, including targeted drug delivery, diagnostic imaging, and biosensing.

This section details the principal techniques employed for the carbohydrate functionalization of SPMNPs. These methods are broadly categorized into covalent and non-covalent approaches. An overview and comparative analysis of these strategies will be provided, referencing foundational work in the field [51].

#### 4.2.1. Covalent Coating Methods

Covalent coating methods are characterized by the establishment of robust chemical bonds between carbohydrate materials and the surfaces of SPMNPs. This approach ensures a stable and permanent linkage that can withstand various physiological and environmental conditions, making it ideal for long-term applications. The most prevalent covalent strategies include:

- Silane Coupling Agents: This method involves using organosilanes (e.g., (3-aminopropyl) triethoxysilane (APTES)) to create a siloxane network on the metal oxide surface of SPMNPs. This layer introduces reactive functional groups (e.g., amines, thiols, or epoxides) that can subsequently form covalent bonds with hydroxyl or amino groups on carbohydrate molecules [134].
- Carbodiimide Chemistry: A widely used strategy for forming amide bonds. Carbodiimide reagents, most commonly 1-ethyl-3-(3-dimethylaminopropyl) carbodiimide (EDC), activate carboxylic acid groups on either the nanoparticle surface or the carbohydrate. This activated intermediate then reacts with primary amine groups on the counterpart to form a stable amide linkage [135,136].
- Click Chemistry: This approach leverages highly efficient and selective reactions, such as the copper-catalyzed azide-alkyne cycloaddition (CuAAC). By functionalizing the SPMNP surface with an alkyne group and the carbohydrate with an azide group (or vice versa), a specific 1,2,3-triazole linkage is formed under mild conditions. This method is valued for its high yield, specificity, and orthogonality to other functional groups [123].
- Aldehyde linker chemistry: This method involves functionalizing the SPMNP surface to introduce aldehyde groups (-CHO). A common approach is to use a bifunctional linker like glutaraldehyde, where one aldehyde reacts with surface amine groups, leaving the other free. This free aldehyde acts as an electrophilic site, reacting with nucleophilic primary amines on carbohydrates to form a reversible Schiff base (-C=N-). This intermediate is then stabilized via reduction (e.g., with sodium cyanoborohydride) to yield a stable, irreversible secondary amine bond, permanently conjugating the carbohydrate [137].

**Figure 3:**
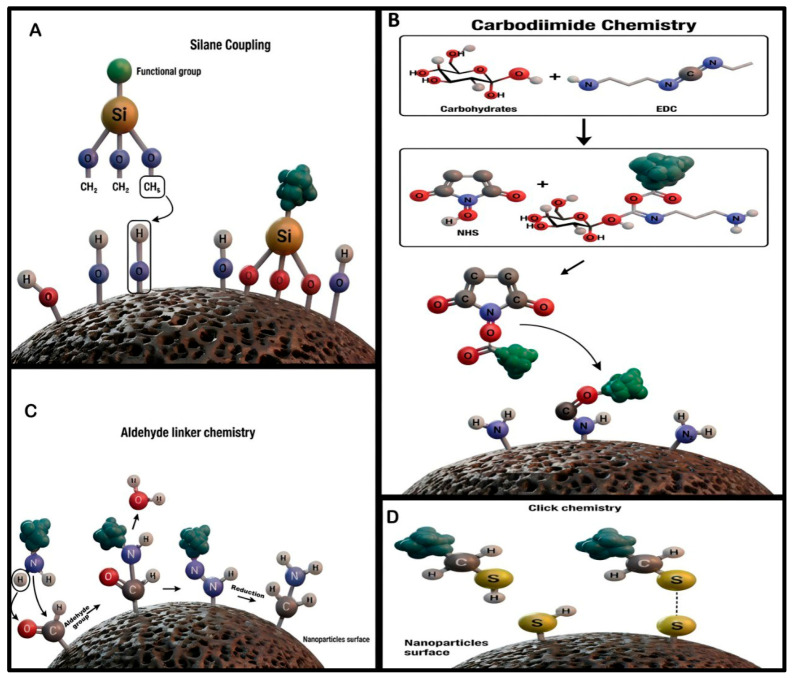
Covalent Strategies for the Surface Functionalization of Nanoparticles with Carbohydrate Materials. (**A**) Silane Coupling: Involves the reaction of a silane reagent, which contains a functional group, with hydroxyl groups on the nanoparticle surface to form a stable siloxane bond; (**B**) Carbodiimide Chemistry: Utilizes EDC and NHS to activate carboxyl groups on carbohydrates, which then react with amine groups on the nanoparticle surface to form a stable amide bond; (**C**) Aldehyde Linker Chemistry: Involves the reaction of an aldehyde group (often from an oxidized carbohydrate) with an amine group on the nanoparticle surface to form an unstable Schiff base, which is then reduced to a stable secondary amine; (**D**) Click Chemistry: Represents a highly efficient and selective reaction, such as the thiol-ene or copper-catalyzed azide-alkyne cycloaddition, used to link functionalized carbohydrates to complementary groups on the nanoparticle surface.

These covalent strategies—Silane Coupling, Carbodiimide Chemistry, Aldehyde Linker Chemistry, and Click Chemistry—provide a versatile toolkit for the robust functionalization of SPMNPs, as illustrated in (Figure 3) [123].

#### 4.2.2. Non-Covalent Coating Methods

Non-covalent coating methods rely on physical interactions—such as van der Waals forces, hydrogen bonding, and electrostatic attraction—to couple carbohydrate materials to the surfaces of SPMNPs. These approaches are generally simpler to implement and can be performed under milder conditions than covalent methods, making them suitable for sensitive biomolecules. Prominent non-covalent strategies include:

- Electrostatic Assembly: This method exploits the attraction between oppositely charged molecules. Carbohydrates can be functionalized or chosen to carry a specific charge, allowing them to bind directly to SPMNPs with an opposing surface charge through electrostatic interactions [138].
- Hydrophobic Interactions: Carbohydrates containing hydrophobic moieties (or those modified with hydrophobic groups) can interact with hydrophobic regions on the surface of SPMNPs. This type of interaction enhances the affinity and stability of the carbohydrate-nanoparticle complex, which is crucial for various applications in biochemistry and material science [138].
- Layer-by-Layer (LbL) Assembly: This versatile technique involves the sequential, alternating deposition of oppositely charged polymers and/or carbohydrates onto the nanoparticle surface. By carefully controlling the deposition cycles, precise multilayer coatings can be constructed, allowing for fine-tuning of the nanoparticle’s properties, thickness, and functionality [139].
- Physical Adsorption: The simplest non-covalent method, physical adsorption, relies on the passive adherence of carbohydrate molecules to the SPMNP surface when mixed in solution. This process is driven by the inherent affinity between the molecules and the nanoparticle surface characteristics, such as roughness or chemical composition [103,140].

**Figure 4:**
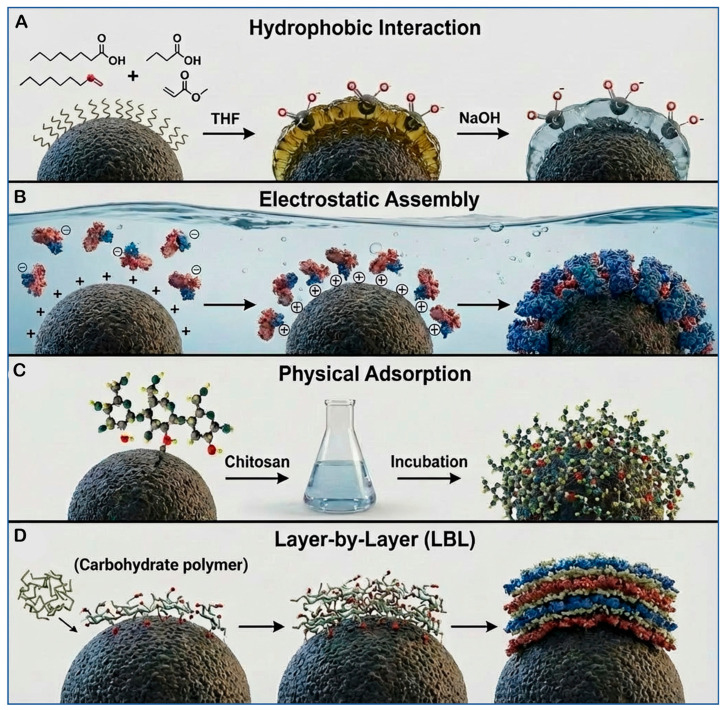
Non-Covalent Strategies for the Surface Functionalization of Nanoparticles with Carbohydrate Materials. (**A**) Hydrophobic Interaction: Involves the self-assembly of amphiphilic molecules onto the nanoparticle surface in an aqueous environment, driven by the exclusion of non-polar groups from water; (**B**) Electrostatic Assembly: Relies on the attractive forces between oppositely charged species, where charged carbohydrate polymers are adsorbed onto a nanoparticle surface with a net opposite charge; (**C**) Physical Adsorption: A general term for the spontaneous binding of carbohydrate polymers (e.g., Chitosan) to the nanoparticle surface through weak forces like van der Waals forces and hydrogen bonding, often followed by an incubation period; (**D**) Layer-by-Layer (LBL): A sequential deposition technique where alternating layers of oppositely charged carbohydrate polymers are adsorbed onto the nanoparticle surface to build a multilayer shell.

In summary, non-covalent methods offer easier and gentler pathways for surface functionalization compared to covalent bonding. Figure 4 provides a visual summary of these key non-covalent assembly procedures: Hydrophobic Interaction, Electrostatic Assembly, Physical Adsorption, and LbL assembly [138,139,140].

#### 4.2.3. Comparison of Covalent vs. Non-Covalent Coatings

Choosing the appropriate coating technique for SPMNPs must be carefully considered after analyzing both properties and appropriateness of either covalent or non-covalent techniques [141].

Covalent coatings are known for their high durability. They provide strong chemical bonds to the surface of the nanoparticle. This property helps the coatings to remain stable even after exposure to harsh conditions like multiple washing cycles, organic solvent treatment, or high or low pH and ionic strength [142]. Such properties make covalent coatings suitable for applications related to *in vivo* drug delivery, targeted therapy, and diagnosing through imaging modalities such as MRI [143].

Non-Covalent Coatings, on the other hand, have a different set of advantages. These coatings, which use weaker physical interactions like van der Waals, hydrogen bonding, and electrostatic binding, are generally faster, easier, and cheaper to produce. Though not as strong as Covalent Coatings, Non-Covalent Coatings have a number of advantages, which make them particularly useful for R&D applications where conditions keep changing [144].

The choice between the two strategies hinges on the specific application requirements. Covalent coating is the preferred solution when a permanent, robust, and chemically stable functionalization is needed, despite often involving more complex synthesis procedures. Conversely, non-covalent coating is the better alternative when simplicity, cost-effectiveness, and easy adaptability or reversibility of the surface layer are prioritized.

### 4.3. Innovative Approaches for the Carbohydrate-Based Coating of SPMNPs

SPMNPs are increasingly recognized for their potential in various biomedical applications such as drug delivery, MRI, hyperthermia therapy. By applying carbohydrate-based coatings to these nanoparticles, their biocompatibility, the targeting capabilities, and the stability within the biological environments can be improved. Below are several novel strategies for the carbohydrate-based coatings of the SPMNPs:

Glycopolymer Coatings: Glycopolymers are specialized polymers that contain the carbohydrate segments as a part of their structure. The various polymerization techniques such as the atom transfer radical polymerization (ATRP) and the reversible addition-fragmentation chain transfer (RAFT) polymerization can be used for their synthesis. The obtained glycopolymers can then be used to modify SPMNPs to enhance their biological activity and target specific cells or tissues [24].

The terminal groups of the glycopolymers can be modified by adding the targeting ligands, such as antibodies or peptides, to enhance the specificity of SPMNPs for designated cells or tissues.

Layer-by-Layer (LbL) Assembly Multilayer Coatings: LbL method enables the systematic and controlled step-by-step deposition of the positively and negatively charged carbohydrate-based materials onto the surface of the SPMNPs. This method offers a precise control over both the thickness and the composition of the multilayer coating, enabling researchers to tailor the properties for specific functional applications. Such a customization is essential to optimize the performance of these materials for the different uses [145].

Bioactive Layering: The integration of the bioactive molecules, like the enzymes or the growth factors, into the carbohydrate layers can enhance the functionality of the nanoparticles by promoting the targeted delivery and the controlled release [146].

Self-Assembled Monolayers (SAMs): Carbohydrate-Functionalized Thiols: Using the thiolated carbohydrates, it is possible to create self-assembled monolayers on the surface of the metallic nanoparticles to produce a uniform and stable coating. This technique enhances the physiological stability and biocompatibility of the SPMNPs.

Controlling Surface Properties: The density and composition of the carbohydrate self-assembled monolayer (SAM) can be adjusted to modulate the interactions with biological systems; this could involve enhancing the cell adhesion or reducing the protein adsorption [147].

Enzymatic Modification: Enzyme-mediated glycosylation entails the use of enzymes, which are the glycosyltransferases, to modify the surface of the SPMNPs by attaching certain carbohydrate groups. This method facilitates the introduction of complex carbohydrates which mimic the natural glycoproteins enhancing the biocompatibility and easier cellular uptake.

Specific Delivery: An enzymatic coating may be tailored to respond to certain biomolecules in the desired setting, thereby increasing the accuracy of the drug delivery [148].

Hybrid Coatings: Integration of carbohydrate coatings with other substances like lipids, proteins, or inorganic elements can lead to hybrid coatings that utilize the benefits of each material. For instance, lipid bilayers can offer a protective layer, whereas carbohydrates can improve targeting capabilities.

Multifunctional Nanoparticles: Hybrid coatings facilitate multifunctionality, enabling SPMNPs to undertake multiple functions concurrently, including imaging, therapy, and targeted delivery [149].

Natural Polysaccharide Coatings: Biopolymers like chitosan, alginate, pectin and hyaluronic acid are also suitable in coating SPMNPs due to their compatibility with the biological systems and due to their natural breakdown. These compounds have the capability of interacting with biological entities well and increasing adsorption or uptake by cells.

Bioactive Features: Numerous natural polysaccharides contain intrinsic bioactive characteristics that can improve the therapeutic effectiveness of the SPMNPs [67,150].

### 4.4. Methods for Functionalizing Carbohydrates with Ligands for Drug Delivery Applications

Carbohydrates play major roles in numerous biological processes. Synthetic methods have been developed to connect different types of ligands and drugs to partially, as well as fully deprotected monosaccharide scaffolds. These also serve in other bio-medically relevant processes such as biocompatibility and biodegradation. A range of conventional and advanced analytical techniques is required to understand the relationship between the form of the synthetic intermediate and the efficiency of the functionalized carbohydrate product [151,152]. These deliver detailed data on the purity and homogeneity of both precursor and final compounds. Bioassays are needed to determine molecular recognition events between the synthesized neoglycocompounds and their putative cellular carbohydrate receptors, and to understand the mechanism of drug delivery mediated by these systems in living organisms. Magnetic or optical guidance control can help to guide a drug to its target cells and to observe the distribution of the neoglyconanoparticles both in time and in space [153].

#### 4.4.1. Covalent Bonding

A covalent bond is generated by a reaction between one or more reactive chemical groups of the NP coating and the oxidized carbohydrate moieties. The key to successful covalent bonding between the NP’s carbohydrate coating and the oxidized carbohydrates moiety on the cell surface relies on several parameters [24,154]. First, the chemistry of reactive groups available on the NP surface should be mild and efficient, involving conditions close to biological conditions. It may be challenging to modify extensively or in a regiospecific manner the carbohydrate coating itself, owing to its labile nature. The carbohydrate coating contains reducing carbohydrate end groups that would react with various chemical groups such as amine, hydroxyl, carboxyl, and mercapto, oriented on the NP surface [155].

Peptide molecules are most commonly used to covalently modify NP surfaces because of the diversity in the amino acid functionalities, but also because of the benefit they provide in structuring the carbohydrate barrier. Longer side chains of amino acids locate the carbohydrate structure far from the NP, whereas amino acids with shorter side chains will decrease the distance between the NP core and the carbohydrate coating [156,157]. The commonly used point of connection between a peptide chain and the magnetic nanoparticles (NPs) surface is cysteine, because the thiol group can bind to metallic surfaces via a strong metal-thiol bond. Many studies have developed procedures for coating the surfaces of magnetic NPs with cysteine-containing peptides. Sialic acid-bearing molecules, such as N-acetylglucosamine or a sialic acid residue, can be linked to the magnetic NP surface using maleimide groups, while mannoside can be attached to the NP surface via NHS groups [158,159].

SPMNPs represent a promising strategy for targeting brain cancer, particularly through the application of EDC/NHS methods to activate carbohydrate surfaces [160]. This activation facilitates the immobilization of various ligands, such as antibodies, thereby improving biocompatibility and targeting efficiency. Additionally, the integration of a magnetic field further enhances the precise localization and effectiveness of these functionalized nanoparticles in targeting cancerous cells [21,161].

#### 4.4.2. Click Chemistry

Click chemistry is a term that refers to a combination of three (or more) fairly ideal functional groups defined as clickable. The criteria are modular, insensitivity to by-products, high conversion yields, regioselectivity, and excellent tolerance to a variety of functional groups [162]. Ideally, the combination of the reaction products should not result in more than one reaction product, should be highly reliable and thermodynamically favorable, producing maximum chemical turnover without the need for by-product removal, workup conditions, metal detection, or protection, and, last but not least, be a stereo-controlled reaction [163]. Such reactions, which follow these criteria, are azide-alkyne 1,3-dipolar, Diels–Alder, the thiol-ene reaction, the Passerini three-component reaction, the Ugi four-component reactions, and radical conjugated addition [164].

#### 4.4.3. Enzymatic Modification

Recently, a new technique has been developed for the modification of lactose in the presence of SPMNPs. This method used immobilized enzymes for the selectivity of 3-O-glycosylation of the acceptor. The method is economically viable due to enzyme reusability and an environmentally friendly room temperature, without harmful chemicals [165]. Moreover, complex formation does not appear to influence the principal enzymological properties of the components. Formation of the respective mannose complex with nanoparticles and crystalline cellulose driven by α(1,3)-mannosyltransferase and α(1,3)-mannansynthase is proposed. The crystal lattice of a saccharide–enzyme complex is stabilized by the hydrogen bonding between the sugar moiety and the catalytic domain of the enzyme. This can play a physical role in the interaction of properties of glycosyltransferases and sugars [166].

#### 4.4.4. Self-Assembly Techniques

Self-assembly provides a very convenient way to prepare stable nanoparticles, maintaining high inner magnetic properties and obtaining an aqueous solution with great resistivity under varying pH conditions, providing nanoparticles with theranostic characteristics. In this type of system, carbohydrate-based derivatives serve not only to give water solubility to the particles by leading modifications in the environment of the magnetite shell on the surface of the nanoparticles, but also play a key role in the self-assembly of the final supramolecular system [167,168]. Self-assembling nanoparticles are a solution for the stabilization of high magnetite content in aqueous solutions. The carbohydrate-based derivatives obtained are grafted onto the surface of the magnetic particles through a simple and fast reaction, avoiding the use of hazardous reagents in the classical synthesis of magnetite nanoparticles, yielding very stable solutions in a pH range from 2 to 10. Water solubility of these magnetic nanoparticles can be extended simply by increasing the carbohydrates chain length. The prepared nanoparticles possess a high relaxivity value that increases with the magnetic concentration of the particles. These carbohydrate-coated nanoparticles show great capacity to be guided by an external magnet and selectively interact with tumor cells [169].

#### 4.4.5. Glycosylation

It has been envisioned that magnetic nanoparticles coated with glycans, or drugs modified with them, can accumulate in the tumor microenvironment, or specifically in tumor cells, in a way similar to how glycan-coated red blood cells act for the purpose of circulation time prolongation [170]. Targeting tumors by affinity glycoparticles can be realized by the application of glycosylated nanoparticles for normal antigens whose function is different in tumor cells than in normal cells. One may also detect tumor antigens overexpressed on the plasma membrane, but this will trigger scavenger cells, such as Kupffer cells in the liver, and may lead to systemic elimination of the nanoparticles [171]. An alternative way of delivering glycosylated nanoparticles to tumor targets is by boron-neutron capture therapy. Firstly, an anti-cancer drug or a cancer-toxic nutrient should be encapsulated in a glycosylated nanoparticle. Then, the nanoparticle should be modified with a boron group which has a structure very similar to the vanadium substituted into some bacterial trehalose disaccharide involving providing resistance to radiation [172,173].

Glycosylated nanoparticles demonstrate binding dependent on both size and surface chemical properties. Receptor-mediated endocytosis of glycosylated nano-systems is the result of a protein–carbohydrate binding event [174]. The basis of lectin–glycan interaction is a complex hydrogen bonding network, including a hydrophobic effect that determines the geometrical structure of the binding site. It was observed in the past that increasing the size of the molecule causes an increase in the binding constant, often accompanied by a shorter lifetime. A less hydrophilic surface that interacts less with the solvent molecule may result in a small desolvation penalty in the binding rate-limiting association process [175].

**Figure 5:**
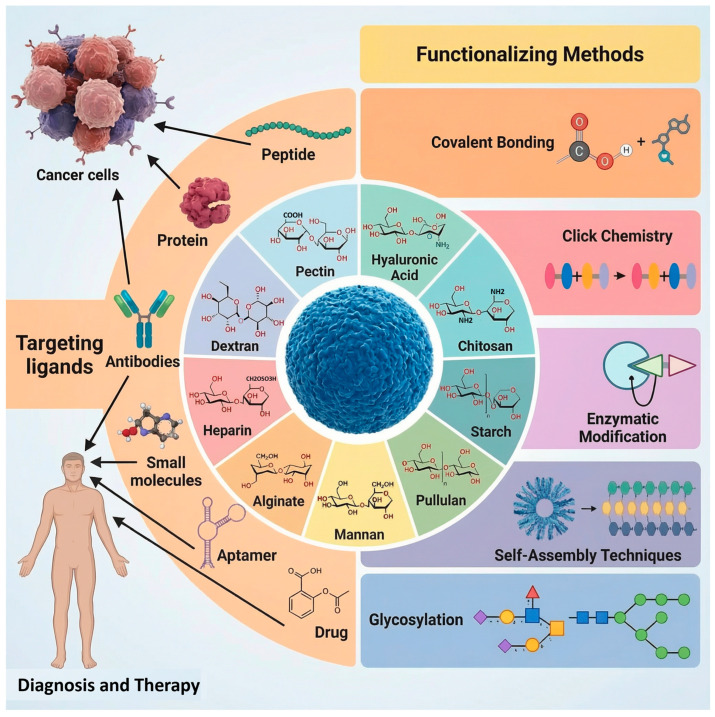
Overview of Carbohydrate-Based Magnetic Nanoparticles: From Polysaccharide Selection and Functionalization to Targeted Drug Delivery Applications.

This review focusing on different versatile methods of chemical attachment to each of these types of low-cost, as well as known medicinal relevance, carbohydrate skeletons (Figure 5) [155,156,157,158,159,160,161,162,163,164,165,166,167,168,169,170,171,172,173,174,175]. These include multi-step strategies to single-step regioselective conjugation methods compatible with drug and nanoparticle stability.

## 5. Magnetic Field Guidance in Targeting Glioma

Navigation of magnetic or magnetized particles inside the human body represents a fascinating scenario for the medical applications, ranging from the drug delivery to hyperthermia treatment and the imaging. Use of magnetically driven engineered particles is emerging as an innovative option to overcome the bottleneck of the BBB, thus bypassing the BBB to deliver the therapeutics to the brain. When the magnetic materials are triggered in the presence of an external magnet, the particles can be guided across the tissues. In the case of the brain malignancies, this property can be exploited to attract particles to the target. In particular, the possibility of guiding a drug-loaded particle to the correct site is challenging, and it is the key to success when such particles convey the therapies across the complex tissue obstacles, such as the BBB and the microenvironment morphology [176]. 

**Figure 6:**
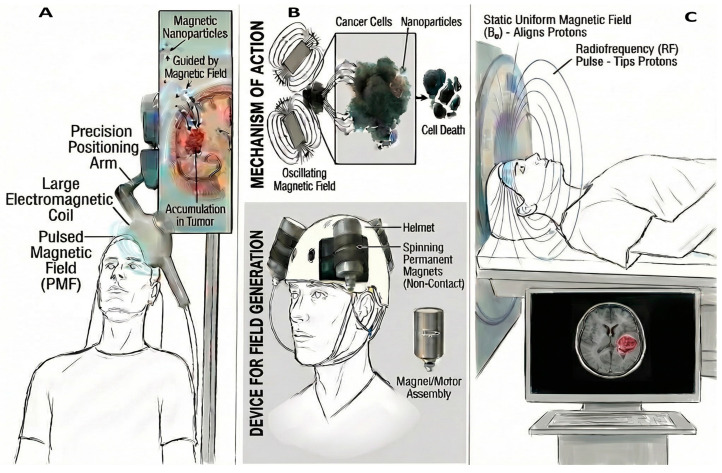
Magnetic Field-Guided Theranostic Magnetic Nanoparticles for Brain Tumors: Utilizing External Fields for Nanoparticle Targeting, Hyperthermia Induction, and MRI Monitoring. (**A**) Nanoparticle Targeting: Magnetic nanoparticles are guided to accumulate in the tumor using a large electromagnetic coil that generates a Pulsed Magnetic Field (PMF), directed by a precision positioning arm; (**B**) Device for Field Generation: A helmet containing spinning permanent magnets (non-contact) or a magnet/motor assembly can be used to generate the necessary magnetic fields for hyperthermia; (Mechanism of Action): Once accumulated, an Oscillating Magnetic Field is applied, causing the nanoparticles to generate heat, leading to cancer cell death; (**C**) MRI Monitoring: The process is monitored using MRI, where a Static Uniform Magnetic Field (B_0_) aligns protons and a Radiofrequency (RF) Pulse tips them, allowing for real-time visualization and assessment of the tumor and treatment efficacy.

Today, SPMNPs in a synergistic combination with MRI imaging are the ideal candidate materials to contrast the MRI and control the particle accumulation, but the navigation of these particles in proximity to a magnetic target is still an open question [36,177]. Unquestionably, the high gradient magnets can be used in hyperthermic treatments by favoring the particle heating through dielectric hysteresis [178]. Close proximity to the iron oxide nanoparticle, and the consequently higher specific absorption rates, makes all nanoparticle heating processes more efficient, and, in the case of the brain, it limits the damage to the healthy tissues. This review considers the possibility of using a magnetic field to drive a large number of particles close to an external target (Figure 6) [108,176,178], i.e., the tumor, focusing on experimental and numerical data on the single iron oxide nanoparticle behavior under the influence of an applied low-frequency traveling magnetic field [1].

### 5.1. Principles of Magnetic Targeting Using External Magnetic Fields

To produce efficient magnetic targeting using external magnets with SPMNPs, the deepest site of the brain cancer mass should be as close as possible to the magnet position [29]. Easy visualization of a tumor is at times possible using MRI and is often complex on T2-weighted imaging due to edema and/or tumor similarity.

Therefore, post-gadolinium T1-weighted imaging could allow a more accurate tumor delineation, but this method is somewhat invasive due to the systemic administration of a contrast agent [179]. This is directly linked to the clinical relevance of localized SPMNPs guidance exiting the blood circulation, favorable to non-targeted cells at some locations, providing low levels of external field. Naturally, the brain monolayer coating of both endothelial cells and astrocytes surrounding the BBB makes this structure efficient against the concentration of SPMNPs in the healthy tissue near tumors [161,180].

An invasive targeting procedure consists of injecting SPMNPs directly into the tumor using positive-pressure stereotactic devices and monitoring the diffusion using MRI guidance. The significant advantage of this method is the high concentration of nanoparticles in the tumor tissue. Post-hypnotic BBB disruption is often used to increase such a concentration that should be enhanced to favor optical imaging or magneto-thermal effect [9].

### 5.2. Mechanisms of Enhanced Accumulation of SPMNPs at Tumor Sites Through Magnetic Guidance

The application of an external magnetic field allows for the precise targeting of SPMNPs to tumor sites. By applying a magnetic field near the tumor area, magnetic nanoparticles can be directed towards the tumor, overcoming the limitations of passive targeting that often leads to poor drug delivery efficiency. The external magnetic field exerts a force on the magnetic nanoparticles, causing them to move towards the region of interest and accumulate at the tumor site [181].

High Retention through the Enhanced Permeability and Retention (EPR) Effect: Tumors typically have an imperfect and permeable vasculature, which supports the extravasation of nanoparticles of the bloodstream into the tumor interstitium. The increase in permeability of such vessels is known as the EPR effect, and it enables the accumulation of SPMNPs to increase. The EPR effect can be further increased by magnetic guidance which can more efficiently target nanoparticles to the tumor microenvironment where they can be held by the disorganized lymphatic drainage [182].

Systemic Clearance Minimization: The systemic circulation time of SPMNPs can be increased with the help of magnetic guidance. This gives a chance to the nanoparticles to take a longer time to get to the tumor site before they are eliminated in the blood. The fact that the nanoparticles are retained in the bloodstream closer to the area of the tumor by the magnetic field ensures that they are less likely to be eliminated by the spleen or liver [181].

High Cellular Internalization: Magnetic guidance can facilitate Cellular Internalization of SPMNPs by facilitating tumor–cell interaction. The external magnetic field might assist to induce nanoparticles to go to the cells expressing the particular surface receptors: At the tumor site, the uptake of the nanoparticles by cancer cells via endocytosis may occur and this is facilitated by the interaction of the specific tumor cell with a certain ligand or antibody attached to the nanoparticles [183].

Improved Distribution in Tumor Microenvironment: Magnetic guidance can be used to more evenly distribute SPMNPs in the tumor. This is more advantageous in heterogeneous tumors where accessibility of some regions by passive delivery can be lower. Nanoparticles can be helped to overcome physical barriers inside the tumor by applying a magnet field, which will allow application of a stronger penetration and distribution inside the tissue [184].

Targeted Drug Delivery: In case SPMNPs are loaded with therapeutic agents, magnetic guidance can be used to maximize the delivery of drugs to tumor cells. The selected method will lessen the amount of exposure of the healthy tissues to the drug and lower side effects. The integration of magnetic targeting and drug-ligand receptor affinity will guarantee the release of drugs in the primary level at the tumor site, which will increase their therapeutic effect [185].

### 5.3. Experimental Studies Demonstrating Magnetic Field-Guided Targeting in Glioma Models

Barbaro et al., developed a unique metal vapor synthesis method to produce glucose-coated SPMNPs that exploit the high glucose requirements of cancer cells. By targeting the GLUT1 transporter, these nanoparticles are selectively absorbed by pancreatic adenocarcinoma cells. This targeted approach offers a promising way to deliver chemotherapeutic drugs directly to tumors, enhancing efficacy and limiting side effects. The study also used different methods of characterization to verify the effective production of nanoparticles. In vitro experiments indicated that glucose coating resulted in increased uptake in GLUT1-positive cells which justified the targeted delivery hypothesis. The results provide the basis of the research on the use of glucose-conjugated nanoparticles as a powerful delivery method in cancer therapy [186].

A current study by Pouyan Kheirkhah et al., contains a good potential solution to the treatment of intramedullary tumors of the spinal cord with the use of magnetic drugs. Through the use of SPMNPs which can be navigated through an external magnetic field, the studies demonstrate a strategy that can possibly promote the concentration of therapeutic agents to tumor sites with minimal side effects to the system. The findings underscore the potentiality of magnetic targeting in the clinical environment especially in addressing tumors that are hard to treat; the experimental procedures involved the production of magnetic nanoparticles and their consequent use in experimental animals that had spinal cord tumors. It was shown that therapeutic outcomes and local drug delivery were measured in the study, which evidences the effectiveness of this targeting strategy. The findings of this study have the potential to make great contributions to the neurosurgery and oncology field as they will offer more effective ways of treating the tumors of the spinal cord [187].

The study by Gabriel N. A. Rego et al., discuss the interactions between magnetic hyperthermia and chemotherapy using aminosilane-coated iron oxide nanoparticles. This is supported by using a combined in vitro and in vivo methodology giving a considerable amount of evidence on the therapeutic potential of this dual-modality treatment. The results emphasize the value of repetitive use of hyperthermia that increases the effect of the treatment on the cells of glioblastoma, which is a malignant type of cancer, by applying alternating magnetic fields; the researchers showed that the nanoparticles have the ability to produce heat locally in the area and hence cause cell death in glioblastoma models. The research implied a comprehensive assessment of treatment parameters and outcomes, which showed a potential of better patient outcomes with the help of such innovative treatment strategy. The findings are especially noteworthy because glioblastoma is still among the most problematic cancers with limited treatment options available to it [188].

In the study by Carlotta Pucci et al., there is a set of hybrids magnetic nanovectors that aim to take advantage of several different mechanisms of causing cells of glioblastoma to die. The combination of both lysosomal membrane permeabilization and chemotherapeutic delivery is a multifaceted approach to cancer treatment, thus noted by the research. The advantage of this strategy is that it improves the efficacy of the therapy besides overcoming the problem of drug resistance that is common with the treatment of glioblastoma; the researchers employed different in vitro models to determine the efficacy of the hybrid nanovectors to induce cell death. The findings showed that the nanovectors were able to disrupt the lysosomal membranes, which resulted in an upsurge in drug availability and efficacy on cancer cells. These future applications of this study indicate that this type of hybrid system may play a vital role in eliminating obstacles leading to the successful treatment of glioblastoma [189].

Beola et al., examine a novel therapy method of glioblastoma multiforme (GBM) through lipid-based magnetic nanoparticles (LMNVs) functionalized with peptide angiopep-2 and loaded with the chemotherapeutic molecule temozolomide (TMZ). The main objective was to improve the therapeutic effects through the combination of localized hyperthermia caused by the use of an alternating magnetic field (AMF) with chemotherapy. The authors prepared the LMNVs and tested their capacity to reach GBM cells, and they were able to show an enhanced penetration through the BBB by transcytosis using receptors. In vitro and in vivo studies have demonstrated that hyperthermia induced by AMF and TMZ together caused a significant enhancement of apoptosis in tumor cells and inhibition of tumor growth in an orthotopic mouse model of GBM, overall, the findings highlight the potential of the multifunctional nanocarriers to improve the drug delivery and the treatment outcomes for the aggressive brain tumors like GBM, suggesting a promising avenue for future cancer therapy [190].

The study by Mo Dan et al., addresses a critical barrier in the brain cancer treatment—the BBB—by investigating how the alternating magnetic fields can enhance the uptake of the iron oxide nanoparticles in BBB models. The findings indicate a novel method to facilitate the drug delivery to the brain, a significant hurdle in treating the brain tumors. This research provides a foundation for future studies focused on overcoming the challenges associated with the BBB permeability, the study conducted experiments to evaluate the nanoparticle uptake in BBB models under the influence of magnetic hyperthermia. The Results indicated that the application of an alternating magnetic field significantly increased the association and uptake of the nanoparticles, suggesting a promising technique for improving the therapeutic delivery to brain tumors. The implications of these findings could lead to breakthroughs in enhancing the effectiveness of the treatments aimed at brain cancer [191].

## 6. Synergistic Effects of Carbohydrate Coating and Magnetic Targeting

The greatest challenge in treating malignant brain cancers is the delivery of therapeutic agents accurately to the elusive tumor cells. SPMNPs, a ferrofluid, can be magnetically guided to the brain tumor cells. However, their natural properties render them unsuitable for therapeutic drug loading, and non-specific interactions with non-targeted normal brain tissue could occur [23].

The present invention discloses a group of specially designed nanoparticles with biocompatible and specific binding carbohydrate-based coatings, which are used for targeting and locally delivering to a malignant brain tumor in the middle of the skull to eliminate the therapies of non-target related normal brain damage and further enhance therapeutic efficacy with magnetic guidance in the future [24,132]. In essence, this development introduces a targeted drug delivery mechanism for brain applications that, when integrated with low-toxicity natural nanoparticles exhibiting potent magnetic characteristics, negates the necessity for external transportation or magnetic assistance in drug therapy. This system aspires to bolster both the efficacy and accuracy of non-invasive molecular imaging techniques for the treatment of brain tumors and magnetic fields [192,193].

The carbohydrate coating is necessary to reduce protein opsonization between the surface of the nanoparticles and proteins. This cover decreases the ability of the reticuloendothelial system to remove them quickly, and passive accumulation occurs through the enhanced permeability and retention effect close to passive GBM targeting [194]. However, the carbohydrate coating is hydrophilic and constantly interacts with water. It can be enhanced by increasing the carbohydrate chain length and therefore increasing the van der Waals forces that are oriented along the carbohydrate chain length. Nonetheless, this approach is still in its infancy, and some questions arise [195].

Carbohydrate hydrophilicity is equal to magnetic susceptibility, which is an aptitude of matter to obtain a net magnetization by the application of a magnetic field. The long carbohydrate chain length enhances their interaction with water but may also decrease their interaction with a magnetic field. Consequently, the carbohydrate-coated magnetic nanoparticles are no longer attracted to a magnetic field because they are no longer water-dispersible. It confirms that an optimal mesoporosity and pore diameter of 30 nm or greater were necessary to achieve good water dispersibility and water-induced colloidal stability. A potential solution for better balancing the carbohydrate hydrophilicity and magnetic field interaction is to go the opposite way, that is to generate high local hydrophilicity in the carbohydrate coating [196].

### Expected Outcomes from Combining Carbohydrate-Based Coatings with Magnetic Targeting Strategies

The effective combination of a magnetic field and the carbohydrate-based functionalized SPMNPs will provide a creative method allowing an accurate therapeutic concentration of toxic drugs to be achieved and any harmed normal tissues shaded from systematic exposure, which is particularly important for the targeted delivery of harmful drugs, especially for the central nervous system therapy. The successfully exploited superparamagnetic iron oxide therapeutic systems designed hereby should bear the following physical and chemical properties with minimum contradiction: the spatially close breakdown of the BBB, enough penetration into the BBB, confinement within the brain lesion, accumulation at the brain lesion, the release of toxic drugs only when a magnetic field is present, and the ability to guide movement by an external magnetic field [9,23]. The innovative combination of physicochemical properties designed for the carbohydrate-based coatings does need to go through a rigorous and appropriate strategy for feasible utilization.

Within the continuously evolving field of nanotechnology, SPMNPs are thought to be potential candidates for wave-based diagnosis and therapy, but there exists a significant challenge in translating these potential candidates to clinical reality in practice, especially for the purpose of central nervous system therapy.

## 7. Challenges and Future Perspective

The emergence of malignant glioblastoma cells in structures crossed by the BBB poses numerous significant challenges to the therapeutic approach. Current concepts of drug delivery at the BBB refer to the equilibrium of passive diffusion and efflux mechanisms. Passive diffusion could be useful in the uptake of essential solubilizing entities accompanying active transport systems. This way, the design and control of new therapeutic drugs warrant equal attention to both diffusion and efflux capabilities [1]. Patients with malignant glioblastoma cells require medication at high doses to overcome the defense from the human BBB [17]. Local application prolongs the effect of the magnetic nanoparticle suspension at the brain tumor site due to the specific activity of an external magnetic field. To avoid logistical and economic limitations, it requires further research employing a series of weaker magnetic fields.

### 7.1. Current Challenges in the Development of Targeted Therapies for Glioma

Glioma is one of the most aggressive and difficult-to-treat brain cancers. The number of drugs able to effectively target tumors is limited by the presence of the BBB and the existence of cancer stem cells, which exhibit resistance to standard treatments. In fact, heterogeneous cell populations and the presence of cancer stem cells in glioma contribute to tumorigenesis, aggressiveness, infiltration, and resistance to chemotherapy and radiotherapy. The main goal of brain cancer therapy remains the removal of the cancer stem cell subpopulation that remains after the administration of chemotherapy and/or radiotherapy. Cell-surface markers of cancer stem cells from a broad range of tumors, not only in glioma, promote resistance to chemotherapy and radiotherapy. In addition, cells that have undergone the epithelial–mesenchymal transition also have chemoresistance and radio-resistance [17,197].

The main requirements for developing targeted therapies to address the heterogeneity and complexity of gliomas result from the intratumoral and intertumoral environment; only therapeutic agents that can adhere to the blood vessels in the tumor microenvironment will reach glioma cells, and the therapeutic properties of these agents will be limited by the BBB. Although many pharmaceutical and nanotechnological approaches have been proposed, there is still no routinely employed therapy that directly targets and selectively kills cancer stem cells and thus selectively inhibits cancer development and recurrence. SPMNPs coated with carbohydrates with known anti-cancer stem cell activity are effective drug carriers not only with increased efficacy, but also with a potential active transport via an external magnetic field [51,193].

### 7.2. Limitations of Existing Methods and Potential Solutions

The standard course of treatment for brain cancer involves surgery to remove cancerous tissue and then follow-up treatments to eliminate any remaining cancer. However, current non-invasive treatments are limited by the BBB and the lack of selectivity and concentration of chemotherapeutic agents within the brain [198]. Nanoparticles, including iron oxide and gold, represent a promising avenue for developing new therapeutic strategies. The BBB tends to facilitate the passage of carbohydrates more readily than various chemotherapeutic agents; therefore, nanoparticles are often perceived as nutrients rather than foreign entities within this pathway. Upon entering the brain, these nanoparticles can selectively attach to receptors present on cancerous cells, enabling them to either generate heat or release therapeutic agents to combat the cancer.

Additionally, when influenced by an external magnetic field, magnetic nanoparticles can effectively traverse the BBB and accumulate at the tumor site [199,200].

### 7.3. Future Directions for Research on SPMNPs, Carbohydrate Coatings, and Magnetic Targeting

SPMNPs have exhibited potential as drug delivery agents in preclinical and clinical studies. The relatively large dimensions and irregular shapes of current groupings of SPMNPs hamper effective crossing of biological barriers, such as the BBB. Carbohydrate-based coatings have the potential to improve immune system evasion by SPMNPs, enhance transport across the BBB, and localize the particles to the desired anatomic site. Specifically, biocompatible, non-immunogenic, and flexible saccharide coatings on SPMNPs have been shown to enhance NP delivery to brain cancer sites and yield significant increases in survival days. Future investigations on the size, shape, and structure of SPMNP groupings would help to identify the optimized parameters that promote SPMNP anatomic targeting, targeting of anatomical structures, and intracellular access and retention. Future studies could explore the structure-function relationship between saccharide coatings and SPMNP targeting to intracranial anatomic sites. New animal models could offer increased insights into the size, shape, and structure of small clusters of highly magnetic nanoparticles delivered to numerous brain anatomic sites. Guidelines provided by promising bio-distribution strategies could offer increased insights for direct local targeting of cancer cells in order to block solid tumor-associated symptoms and signs. Dietary carbohydrate sources could be leveraged to provide easily accessible saccharides for research on structural modifications that might enhance SPMNP-cancer cell molecular targeting at intracranial anatomic sites. Finally, the use of dietary saccharides could offer cost-effective options to support translational validation studies.

## 8. Conclusions

The effective treatment of brain cancer critically depends on the ability to deliver therapeutic agents across the BBB to specific tumor sites. Systemic delivery methods are often inadequate due to the inherent risk of neurological damage from non-targeted drugs and the poor magnetic performance of many conventional drug carriers, which hinders their use in magnetic targeting strategies.

Consequently, the development of magneto-controllable drug carriers represents a pivotal advancement. The foundational step for this strategy is the successful testing of SPMNPs in well-established rodent brain cancer models. Early pioneering work has already demonstrated the remarkable potential of this approach, showcasing observable magnetic targeting and “gliding” effects in both brain and subcutaneous glioblastoma models. These studies underscore a core conclusion: the precise specification of magneto-controllable carriers holds immense potential for achieving patient-specific therapeutic success with high precision and minimal off-target effects. This is enabled by their dual functionality as MRI-trackable imaging agents and as magnetically guided vehicles for controlled drug release.

The primary aim of this review was to highlight the rapid recent progress in developing carbohydrate-coated SPMNPs. We have detailed advances in the design and synthesis of carbohydrate-coated magnetic nanoparticles, emphasizing the fundamental necessity of creating aqueous-dispersible, biocompatible systems. Crucially, these advances center on the strategic modification and conjugation of the carbohydrate coat with high-affinity targeting ligands to achieve active cellular homing. This involves leveraging the carbohydrate as a versatile scaffold for attachment, utilizing advanced chemical techniques such as covalent bonding and Click Chemistry to immobilize ligands like peptides and antibodies. These resulting glycoconjugates are specifically designed to exploit the altered glycosylation patterns and overexpression of specific lectins on the surface of brain cancer cells, thereby ensuring precise cellular recognition and uptake. These coatings are crucial for ensuring that drug-loaded magnetic particles are fully MRI-locatable and trackable under an applied magnetic field.

While the chemistry of these carbohydrate coatings is complex, rich, and not yet fully elucidated—as they exhibit variable efficiency across different settings—their most promising application is clear: the magnetic field-guided transport of BBB-permeable drugs to brain tumors. Among the various candidates, carbohydrate-coated magnetic iron oxide nanoparticles emerge as particularly promising leaders in this field, and merit significant further research and development to realize their full clinical potential.

## Figures and Tables

**Table 1:** Strategies for Polysaccharide-Coated MNPs in Imaging.

Polysaccharide	Modification/Cargo	Application Context	Model System	References
Alginate	APA	Myoblast cell line study	C2C12 myoblast cell line (in vitro)/Mice abdominal cavity (in vivo)	[68]
Chitosan	CTX, PEG	Drug delivery for brain tumors	ND2: SmoA1 mice (in vivo)	[69]
Dextran	FITC-derivatized Tat peptide	Targeting hematopoietic and neural progenitor cells	Hematopoietic and neural progenitor cells (in vitro)	[70]
Hyaluronic Acid	Dopamine	Cell line studies for drug efficacy	HCT116 and NIH3T3 cells (in vitro)	[71]
Heparin	Gold-deposited glycol chitosan	Anti-tumor therapy	SCC-7 tumor-bearing mice (in vivo)	[72]
Mannan	Carboxylic	Subcutaneous injection delivery	Rat model (in vivo)	[73]
Pectin	Curcumin-loaded	Anti-cancer therapy	Various cancer models (in vitro/in vivo)	[74]
Pullulan	Hyperthermic effect	Hyperthermia studies	L929 and KB cells (in vitro)	[75]
Starch	PEG	Drug delivery for brain tumors	Male Fisher 344 rats with 9L-glioma brain tumors (in vivo)	[76]

Alginate-Poly-L-lysine-alginate (APA), Chlorotoxin (CTX), Fluorescein Isothiocyanate (FITC), squamous cell carcinoma (SCC-7).

**Table 2:** Properties of Commonly Used Polysaccharides.

Polysaccharide	Origin Type	Natural Source	Electrical Charge	Defining Functional Groups	References
Alginate	Plant/Algae	Brown algae	Negative	Hydroxyl (OH), Carboxyl (COOH)	[68]
Chitosan	Animal	Shrimp and crustacean exoskeletons	Positive	Hydroxyl (OH), Amine (NH_2_)	[69]
Dextran	Microbial	Microbial product (e.g., wine)	Neutral	Hydroxyl (OH)	[70]
Hyaluronic Acid	Animal	Connective, epithelial, and neural tissues	Negative	Hydroxyl (OH), Carboxyl (COOH)	[71]
Heparin	Animal	Animal tissues	Negative	Hydroxyl (OH), Sulfated Hydroxyl (OSO_3_H)	[72]
Mannan	Plant	Plant polysaccharide (storage)	Neutral	Hydroxyl (OH)	[73]
Pectin	Plant	Fruits (especially citrus and apples)	Negative	Carboxyl (COOH), Hydroxyl (OH)	[74]
Pullulan	Microbial	Produced from starch by Aureobasidium pullulans	Neutral	Hydroxyl (OH)	[75]
Starch	Plant	Energy storage in green plants	Neutral	Hydroxyl (OH)	[76]

## Data Availability

Data is contained within the article.

## Abbreviations

The following abbreviations are used in this manuscript:

BBB	Blood-brain barrier
CNS	Central nervous system
EDC	1-Ethyl-3-(3-dimethylaminopropyl) carbodiimide
MRI	Magnetic Resonance Imaging
NHS	N-Hydroxysuccinimide
NP	Nanoparticle
SPMNPs	Superparamagnetic iron oxide nanoparticles
TMZ	Temozolomide
GSC	Glioblastoma stem cell
LBL	Layer-By-Layer
